# Interactions of Antibacterial Naphthoquinones with Mesoporous Silica Surfaces: A Physicochemical and Theoretical Approach

**DOI:** 10.3390/ph15121464

**Published:** 2022-11-25

**Authors:** César Iván Corpus-Mendoza, Denisse de Loera, Lluvia Itzel López-López, Brenda Acosta, Sarai Vega-Rodríguez, Gabriela Navarro-Tovar

**Affiliations:** 1 Facultad de Ciencias Químicas, Universidad Autónoma de San Luis Potosí, Manuel Nava 6, Zona Universitaria, San Luis Potosi 78210, Mexico; 2 Instituto de Investigación en Zonas Desérticas, Universidad Autónoma de San Luis Potosí, Del Altair 200, del Llano, San Luis Potosi 78377, Mexico; 3 Coordinación para la Innovación y Aplicación de la Ciencia y Tecnología, Universidad Autónoma de San Luis Potosí, Sierra Leona 550, Lomas de San Luis, San Luis Potosi 78210, Mexico; 4 Consejo Nacional de Ciencia y Tecnología, Insurgentes Sur 1582, Crédito Constructor, Benito Juárez, La Ciudad de Mexico 03940, Mexico

**Keywords:** 1,4-naphthoquinones, SBA-15 particles, isotherm models, density functional theory, drug-delivery vehicles

## Abstract

1,4−naftoquinone (NQ) molecules have been extensively evaluated as potent antibacterial compounds; however, their use is limited, since they have low water solubility and exhibit toxicities in healthy eukaryotic cells. A possible path to overcoming these challenges is the use of particulate vehicles, such as SBA-15, which is a biocompatible and biodegradable mesoporous silica material, that may enhance drug delivery and decrease dosages. In this work, an isotherm model-based adsorption of three NQs into SBA-15 microparticles was evaluated. Interactions between NQs and SBA-15 microparticles were modeled at the B3LYP/6-31+G(d,p) level of theory to understand the nature of such interactions. The results demonstrated that the adsorption of NQ, 2NQ, and 5NQ into SBA-15 fit the Freundlich adsorption model. According to theorical studies, physisorption is mediated by hydrogen bonds, while the most stable interactions occur between the carbonyl group of NQ and silica surfaces. Both experimental and theoretical results contribute to a deeper understanding of the use of SBA-15 or similar particles as nanovehicles in such a way that NQs can be modified in carbonyl or C3 to enhance adsorptions. The theoretical and experimental results were in accordance and contribute to a deeper understanding of how interactions between NQ-type molecules and SiO_2_ materials occur.

## 1. Introduction

Antimicrobial resistance is a global health problem declared by the World Health Organization (WHO) as among the top 10 global public health threats. WHO indicated in 2019 that 25 countries had reported bloodstream infections due to methicillin-resistant *Staphylococcus aureus*, and 49 countries provided data on bloodstream infections due to *Escherichia coli* resistance to third-generation cephalosporins. Moreover, antibiotic-resistant *Mycobacterium tuberculosis* strains limit the treatment of the global tuberculosis epidemic [1]. While the development of new molecules with potential antimicrobial action is slow, the rate of resistance development is always greater. Of the antibiotic-resistant pathogens listed by WHO, only 32 had been tested by 2019, and only 6 were considered innovative. The search for novel antibiotics considers the isolation and chemical modification of secondary metabolites from natural sources, such as 1,4−naphthoquinones (NQs). Our research group has been working extensively on synthesizing novel naphthoquinone derivatives as potential antitumoral and antibiotic drugs. A quinone molecule derived from a naphthalene ring, 1,4−naphthoquinone (NQ), exhibits antimicrobial [2,3,4], antifungal [5], antioxidant [6,7,8], and antitumor activities [9,10,11]. NQs and several derivatives have been isolated from plants, fungi, and animals, and they induce the production of reactive oxygen species (ROS) by redox reactions, which are related to the antibacterial effect against prokaryotic [12] and eukaryotic cells and can trigger apoptosis in tumor cell lines via ROS-modulated signaling pathways [13,14]. However, NQ derivatives possess low solubility in water and they may induce toxic effects in animals [15], and for that reason, using nanomaterials and micromaterials as drug-delivery vehicles can overcome solubility and toxicity problems. The use of nanomaterials and micromaterials offers advantages, such as the protection of drug degradation in living organisms, higher affinities for the drug target site, greater surface area to increase the number of drug molecules onto the vehicle, and potential contributions to synergistic effects [16,17]. Among several materials, mesoporous silica (SiO_2_) particles, such as SBA-15, have been widely studied as drug-delivery vehicles, taking advantage of their large specific surface area, the ease of modifying their chemical nature, and their high biocompatibility and biodegradability [18,19,20,21,22]. SBA-15 (Santa Barbara amorphous) particles were first obtained by Zhao et al., who reported a material with two-dimensional well-ordered hexagonal structures, large pores, and large surface areas [23]. In the last two decades, the biological applications of SBA-15 focused on drug delivery [24,25], vaccine platforms [26,27], and tissue engineering [28,29] among other applications.

Interactions between SiO_2_ materials and organic molecules of biological interest have been studied using different computational chemistry techniques ranging from modeling realistic molecular systems to the energy involved in the interaction. In this sense, some authors proposed molecular modeling methodologies that allow obtaining, in the first instance, realistic models of SiO_2_. For example, in their work, Ugliengo et al. (2008) proposed the construction of surface models for amorphous silica, starting from the dimensional replication of a unit cell of alpha cristobalite and subsequent saturations with hydroxyl groups [30]. On the other hand, the density functional theory (DFT) allows us to study and evaluate molecular structures, intermolecular interactions, and their associative energies, both for molecules of biological interest [31] and nanomaterials, with an adequate level of chemical precision [32]. Moreover, the combination of DFT and other methodologies, such as the quantum theory of atoms in molecules (QTAIM) [33], has allowed the estimation of the energy of H-bonds [34]. These methodologies have been used to study interactions between SiO_2_ materials and anticancer drugs [35,36], such antibiotics as ampicillin [37] and β_1_-receptor blockers [38], as well as other supports used in drug delivery, such as polyethylene glycol (PEG) xerogels [39] and chitosan [40].

Currently, there is limited information on drug molecule interactions with mesoporous silica particles. Nonetheless, the understanding of the physical–chemical interactions between molecules, such as NQs, and the inorganic surfaces of silica oxides may contribute to tuning particle surfaces or modifying NQ structures.

In this work, we aimed to evaluate the interaction of NQ molecules with the surface of SBA-15 particles These interactions were analyzed via experimental and computational studies using DFT and topology analyses with the quantum theory of atoms in molecules (QTAIM).

## 2. Materials and Methods

### 2.1. Chemicals

1,4−naphthoquinone molecules (NQs): 1,4−naphthoquinone (NQ), 2−hydroxy−1,4−naphthoquinone (2NQ), 5−hydroxy 1,4−naphthoquinone (5NQ), and SBA-15 particles were purchased from Sigma-Aldrich (St. Louis, MO, USA). All chemicals were used as received without further purification.

### 2.2. Silica Particle Characterization

The hydrodynamic diameter and Z-potential of the prepared colloidal solutions (100 μg mL^−1^) were measured by Zetasizer Nano ZS90, (Malvern Instruments, Instruments Worcertershire, Worcertershire, UK). The measurements were performed at 25 °C, in triplicate, using disposable polystyrene cuvettes. TEM micrographs were taken with a JEM-JEOL-2100 microscope. The samples were suspended in ethanol and deposited dropwise onto formvar–carbon grids. We used ImageJ v1.53o to measure the length, width, and pore width of SBA-15 particles [41,42]. XRD (X-ray powder diffraction) patterns were measured in a Panalytical EMPYREAN diffractometer using CuKα (λ = 1.54184 Å). The interval of XRD analysis was 5–60° 2θ, with a step size of 0.01° and 1 s of measure time for each step. The diffractograms were analyzed using the X’Pert High Score software [43]. Nitrogen adsorption/desorption experiments were carried out with in a TriStar II-3020 (Micrometrics) device at 77 K. Before analyses, the samples were treated in a vacuum (10^3^ torr) at 300 °C for four hours using a Micrometrics VacPrep 061−Sample degas system. The specific surface area was estimated using the Brunauer Emmet Teller (BET) model. Pore size distributions were calculated using the Barrett–Joyner–Halenda (BJH) model. The average pore size and volume were estimated based on the desorption branch of each isotherm of nitrogen.

### 2.3. NQs Adsorption Measurements

An initial suspension of SBA-15 particles 50 μg in deionized water and NQ, 2NQ, or 5NQ (50, 100, and 150 μg L^−1^) was placed at room temperature and constantly stirred. For adsorption isotherms studies, SBA-15 particles were immersed in NQs solutions at different concentrations (5–150 mg L^−1^) and stirred for 24 h, which is sufficient time for the mixture to reach equilibrium (data not shown). After reaching equilibrium, samples were filtered and measured by UV-Vis spectrophotometry; the filtrate was immediately analyzed using a Jenway 7305 UV-Vis spectrophotometer (200–700 nm) at room temperature to determine NQ’s free concentrations. The equilibrium adsorption of NQs (*q_e_* = mg NQs per gram of silica) was calculated by Equation (1):

(1) $$q_{e}=\frac{\left(C_{0}-C_{e}\right)V}{m}$$

where *C*_0_ and *C_e_* (mg L^−1^) are the initial and equilibrium concentration, respectively, of any of the three NQs in solution. *V* (L) is the volume of NQs solution, and *m* (g) is the mass of the adsorbent SBA-15 particles used in the experiment. The fit of the obtained experimental data to different adsorption isotherm models can be performed using their linear forms after a mathematical transformation of the values as appropriate [44]. However, other authors suggest that such treatments increase the error of the parameters obtained in each model [45,46,47]. In this work, the adsorption isotherm data were fitted to Langmuir, Freundlich, and Temkin isotherm equations in their non-linear forms. Since the methodology for fitting the data by non-linear regression employs the minimization of an error function from the starting parameters, we used the parameters obtained from the fit to the linear form of each model as the starting parameters in each case.

### 2.4. Spectroscopic Characterization of SBA-15 and NQs

The interaction between SBA-15 particles and NQs was studied by recording infrared spectra. The spectra were collected in the ATR mode using an FT-IR spectrometer (Thermo Scientific NICOLET iS10-ATR, Waltham, MA, USA) operating at 1 cm^−1^ resolution across the 400–4000 cm^−1^ range.

### 2.5. Theoretical Approach

NQ, 2NQ, and 5NQ structures underwent atom-by-atom construction using Gauss View 5.0 [48] as a reference to the NQ structures reported in the literature [49]. To simulate a structure that is similar to SBA-15, we constructed a cluster of SiO_2_. The SiO_2_ cluster starts from an α-cristobalite crystal model reported by [50]. To obtain a reactive region, we take the crystallographic plane with the (111) Miller indices using the atomistic simulation environment (ASE) from the Python library [51]. Subsequently, we replicate this surface model two times in the “x” and “z” directions. Finally, we saturate broken bonds with the hydroxyl groups. This cluster is representative of a neutral surface with adsorption sites as it exhibits oxygen atoms, hydroxyl groups, or silicon atoms. Since the DFT has been used for the study of drug adsorptions onto SBA-15 [30], we evaluated the nature of NQ adsorptions onto SBA-15 with the B3LYP hybrid functional [52] in conjunction with the basis set 6-31+G(d,p) [53,54]. The geometrical optimization of all molecules was performed with the B3LYP/6-31+G(d,p) method, and their vibrational frequencies were calculated at the same level of theory to ensure that the optimized geometry corresponds to a minimum on the potential energy surface. Gaussian 09W software was utilized for all calculations [55]. The adsorption of NQs onto SBA-15 particles was modeled using a cluster approach assuming that the NQ− and SiO_2_−optimized models had 1:1 interactions. The resulting models were optimized at the same level of the theory. The QTAIM topology analysis was realized to determine the nature of the intermolecular interaction. QTAIM analyses were carried out using Multiwfn software (free access software, Beijing Kein Research Center for Natular Sciences, Beijing, China) [56].

## 3. Results

### 3.1. TEM Images of SBA-15 Particles

**Figure 1:**
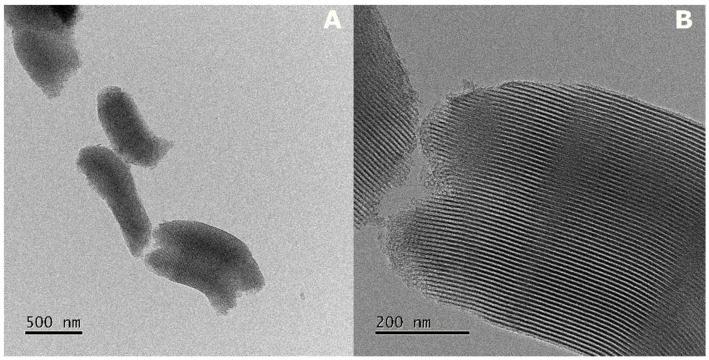
Morphology of SBA-15 nanoparticles. (**A**) TEM image of the irregular shape of particles. (**B**) TEM image of a particle with ordered channels throughout its entire structure.

Figure 1A shows a typical TEM image of the as-received SBA-15 particles. An irregular shape structure was found with a transverse diameter of 403 ± 115 nm and a longitudinal length of 965 ± 247 nm. Furthermore, particles exhibit a series of parallel and ordered channels with a pore width of 5.20 ± 0.88 nm (Figure 1B). Once a morphological characterization was conducted, isotherm adsorption experiments were carried out to analyze possible NQs interactions, and any evidence of interactions is presented first; then, the isotherm models are presented.

**Figure 2:**
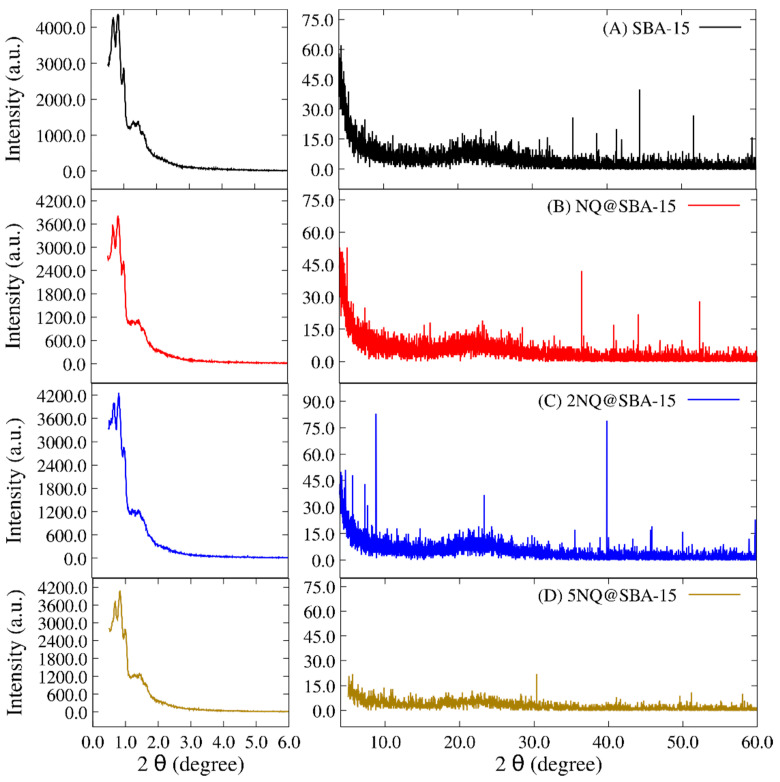
XRD patterns of SBA-15 loaded with NQs. (**A**) SBA-15, (**B**) NQ@SBA-15, (**C**) 2NQ@SBA-15, and (**D**) 5NQ@SBA-15.

In this sense, the XRD diffractograms of SBA-15 alone (Figure 2A, as reference), NQ@SBA-15 (Figure 2B), 2NQ@SBA-15 (Figure 2C), and 5NQ@SBA-15 (Figure 2D) were compared. SBA-15 appears to be a semicrystalline structure with some reflections observed at the 41.2, 44.3, 51.7, and 59.9 values of 2θ degrees corresponding to (012), (200), (112), and (211) of the Miller index; these observations are supported by other reports [57,58] where SBA-15 and similar structures have been studied. In addition, reflections indicate an ordered hexagonal pore array in the range 2θ = 0.5 to 2.

These reflections remain constant with different intensities in the NQ@SBA-15, 2NQ@SBA-15 and 5NQ@SBA-15 systems. However, 2NQ@SBA-15 exhibits three reflections at 8.8, 23.4, and 39.8 values of 2θ, evidencing secondary new crystalline planes when 2NQ is incorporated. On the other hand, 5NQ@SBA-15 (Figure 2D) presents two phenomena: (1) the attenuation of all diffraction bands and (2) a decrement in the overall values of 2θ. The last suggests that the 5NQ incorporation modifies the surface material’s deformation; as a consequence, a loss of semicrystalline domains occurs. This is more evident in the calculated crystallite size values (Table 1) using the Scherrer equation [59,60]. The crystallite size of SBA-15 (911.23 Å) decreases when the particles interact with any of the three NQs; however, in the 2NQs, the new crystalline planes increase the crystallite’s size. Hence, 2NQ has more interactions with SBA-15 particles.

### 3.2. FT–IR of NQ, 2NQ, and 5NQ onto SBA-15 Particles

**Figure 3:**
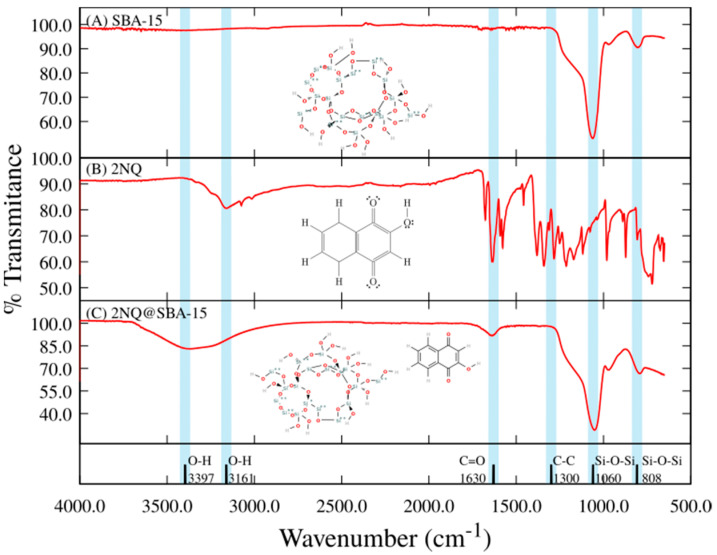
FTIR spectra from top to bottom: (**A**) SBA-15, (**B**) 2NQ, and (**C**) 2NQ@SBA-15.

To continue the evaluation of interactions between SBA-15 and NQs molecules, the FT-IR spectra of naphthoquinone derivatives adsorbed on SBA-15 particles were obtained. To represent these interactions, 5NQ, SBA-15, and 5NQ@SBA-15 are presented in Figure 3. NQ, NQ@SBA-15, 2NQ, and 2NQ@SBA-15 can be found in the Supplementary Materials (Figures S1 and S2) section with similar results. The characteristic peaks obtained in the IR spectrum of SBA-15 (Figure 3A) were at 1060 cm^−1^ and 808 cm^−1^ when assigned to asymmetric and symmetric Si-O-Si stretching, respectively. On the other hand, the IR spectra obtained from the three naphthoquinone derivatives show the following typical absorption bands: 1656 cm^−1^ stretching of the carbonyl functional group C=O, 1603 cm^−1^ corresponding to C=C stretching, 1300 cm^−1^ corresponding to C-C stretching, and 766 cm^−1^ corresponding to out-of-plane flutter. The IR band’s designation for SBA-15 in this work is according to the IR information that is widely reported in the literature [61,62,63]. On the other hand, the IR spectrum corresponding to 5NQ (Figure 3B) shows a band in the region of 3161 cm^−1^ and 3060 cm^−1^ assigned to O-H stretching. Then, the 5NQ interaction on SBA-15’s surface is observed in the blue regions in Figure 3. First, Si-O-Si stretches the characteristic of SBA-15 at 1060 cm^−1^, and 808 cm^−1^ remains, but the intensity decreased in both bands. Then, a broad and flattened band at 3366 cm^−1^ was assigned to O-H and C-H stretches. One more band at 1637 cm^−1^ corresponding to the C=O stretch of NQs appeared in 5NQ@SBA-15. For NQ@SBA-15 and 2NQ@SBA-15, the O-H and C-H stretches appear at 3397 cm^−1^ and 3366 cm^−1^, respectively, and the C=O stretch appeared at 1630 cm^−1^ and 1636 cm^−1^ (Figures S1 and S2).

The changes in intensity and new IR corresponding to the NQs in NQ@SBA-15, 2NQ@SBA-15, and 5NQ@SBA-15 correlate with interactions. The use of IR for following interactions between NQ and SBA-15—like structures have been reported, and the designation of bands in this work was supported by the information previously reported by our group [64,65] and by other research groups [57,66].

Additionally, in Table 1, the hydrodynamic diameter (d.h.) is reported for SBA-15, NQ@SBA-15, 2NQ@SBA-15, and 5NQ@SBA-15. The SBA-15 d.h. value is 618 nm. All three NQs modify the d.h.; however, NQ and 5NQ have increments with values up to 400% and 600%, respectively, suggesting particle aggregation, while the d.h. value for 2NQ is 667 nm (no particle aggregation). These findings correlate with the pZ values (Table 1) where the values are 22.97, 25.17, and 28.4 mV for 5NQ, NQ, and 2NQ, respectively. Values of absolute 30 mV or higher are related to stable and non-aggregated colloids [67,68]; in these systems, 2NQ@SBA-15 is the closest value to the reference. Thus, 2NQ may interact with SBA-15’s surface more effectively, but NQ and 5NQ may promote either particle–5NQ–particle interactions or aggregations between NQ molecules and later depositions onto SBA-15 particles.

**Figure 4:**
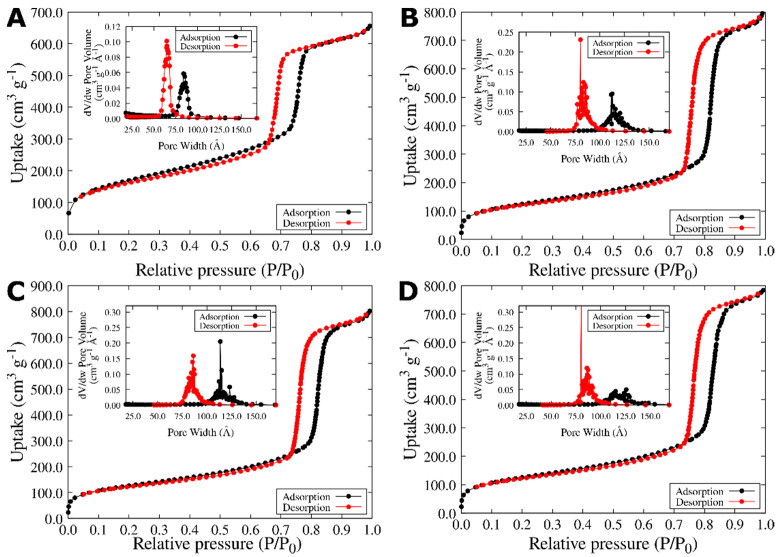
Experimental N_2_ adsorption/desorption isotherms of (**A**) SBA-15, (**B**) NQ@SBA-15, (**C**) 2NQ@SBA-15, and (**D**) 5NQ@SBA-15.

The textural properties of SBA-15 and NQs@SBA-15 were analyzed by N_2_ thermal adsorptions. The obtained data are shown in Figure 4. According to the BET model, SBA-15 was characterized by the adsorption–desorption isotherm (type IV) that is typical for mesoporous materials with a type H1 hysteresis loop [69,70] and a surface area of 597.25 m^2^ g^−1^. In addition, the pore diameter’s distribution curve presented mesopores with an average diameter of 9.7 nm (see insertion in Figure 4A). Note that these data correlate well with those reported by other authors for commercial SBA-15 [71,72].

The attachment of NQs onto the SBA-15 surfaces notably affected the surface area and the pore size’s distribution (Table 1, Figure 4). The surface area for SBA-15 was 597.25 m^2^/g, while the values diminished to 434.88, 441.7, and 438.99 m^2^/g for NQ@SBA-15, 2NQ@SBA-15, and 5NQ@SBA-15 samples, respectively. The decrease in surface area values was attributed to the partial blocking of SBA-15’s surface by the adsorbed molecules.

As can be seen, the interaction between NQ, 2NQ, and 5NQ and SBA-15 modifies the surface of the particles. Given the chemical structure of the NQs, these interactions could be mediated by the H-bonds between the carbonyl and hydroxyl groups of the NQ molecules and silanol groups of SBA-15’s surface. Both 2NQ@SBA-15 and 5NQ@SBA-15 surface area values were slightly higher due to the presence of the hydroxyl group in the 2NQ and 5NQ molecules; then, intermolecular interactions were promoted. Considering the above, we would expect that, as a result, 2NQ and 5NQ were deposited onto the SBA-15, while NQ preserves dispersion. Indeed, the pore size for the NQs@SBA-15 samples increased as the surface area decreased, accompanied by broadening pore diameter´s distribution curves.

### 3.3. Adsorption Isotherm Models of NQ Derivatives on SBA-15 Particles

The adsorption process has been widely described as a phenomenon that is dependent on the physicochemical features of the adsorbent’s surface, the characteristics of the adsorbate, and the chemical environment in which it develops. Different authors proposed a wide range of mathematical models that attempt to describe these adsorption processes. As a result, the number of adsorption isotherm models available is extensive. However, both the Langmuir model and the Freundlich model generally and adequately represent the adsorption phenomenon and model adsorption. This work evaluated the isotherm models with NQ relative to SBA-15.

#### 3.3.1. Langmuir Isotherm Model

The Langmuir [73,74] adsorption isotherm assumes that there are active sites on the surface of the adsorbent. On these active sites, the adsorption process occurs following some features: (1) Each active site is identical in terms of the probability of adsorbing or not adsorbing a molecule; (2) each site can adsorb only one adsorbate molecule; (3) all sites are sterically and energetically independent in their adsorption capacity; i.e., it is not conditioned by the occupation of nearby active sites. The Langmuir isotherm model is described by Equation (2):

(2) $$q_{e}=\frac{Q_{0}bC_{e}}{1+bC_{e}}$$

where $C_{e}$ is the concentration of NQ at an equilibrium (mg L^−1^), $Q_{0}$ is the monolayer adsorption capacity (mg g^−1^), and *b* is the Langmuir isotherm constant related to the energy of adsorption (L mg^−1^). The parameter values of this model can be determined using a linear equation, which is expressed in Equation (3).

(3) $$q_{e}=Q_{0}-\frac{q_{e}}{bQ_{0}C_{e}}$$

#### 3.3.2. Freundlich Isotherm Model

The Freundlich [75] isotherm model describes the adsorption process as a phenomenon in which the adsorbate is not limited to forming a monolayer on the surface of the adsorbent but rather can form multiple layers with a heterogeneous distribution across the entire surface of the adsorbent material. The Freundlich mathematical model is expressed in Equation (4):

(4) $$q_{e}=k_{F}C_{e}^{1/n}$$

where $k_{F}$ is the constant of Freundlich isotherm, which is an indicator of adsorption capacity (mg g^−1^). n is the adsorption intensity. The values of n could indicate the favorability of sorption, where *n* < 1, 1 < *n* < 2, and 2 < *n* < 10 represent poor, moderately difficult, and favorable adsorption conditions, respectively [76]. The linear expression of this model is given by Equation (5).

(5) $$log\left(q_{e}\right)=log\left(k_{F}\right)+\frac{1}{n}log\left(C_{e}\right)$$

By plotting $log\left(q_{e}\right)$ versus $log\left(C_{e}\right)$, the values of $k_{F}$ and n can be determined from the slope and intercept of the plot.

**Figure 5:**
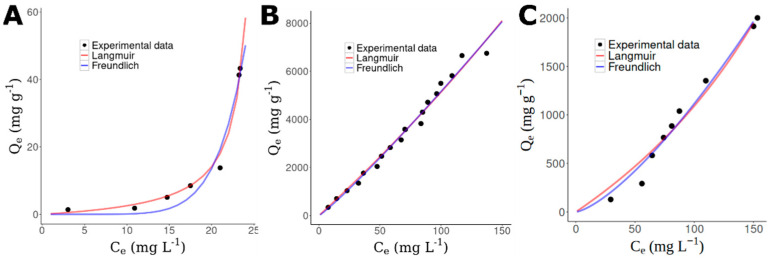
Adsorption isotherms of the (**A**) NQ@SBA-15, (**B**) 2NQ@SBA-15, and (**C**) 5NQ@SBA-15 systems fitted to Langmuir (red) and Freundlich (blue) models.

Thus, using the experimental data of NQ@SBA-15, 2NQ@SBA-15, and 5NQ@SBA-15, Figure 5 shows the fitting for the Langmuir and Freundlich’s models in their adjustments to non-linear equations.

#### 3.3.3. Temkin Isotherm Model

On the other hand, Temkin et al. [77] proposed a model that explicitly considers the interactions between the adsorbate and adsorbent. This model assumes that the heat of the adsorption process depends on the temperature of all molecules in an adsorption layer; the adsorption should decrease linearly rather than logarithmically. The mathematical expression that represents this model is given by Equation (6):

(6) $$q_{e}=\frac{RT}{b_{T}}log\left(A_{T}C_{e}\right)$$

where R is the gas constant (8.314 J K^−1^ mol^−1^), *T* is the temperature (K), $b_{T}$ is the Temkin isotherm constant, and A is the equilibrium binding constant (L g^−1^). A plot of $q_{e}$ versus $log\left(C_{e}\right)$ enables the determination of the isotherm constant.

We include the evaluation of this model to explore the possibility that NQs and SBA-15 interact mainly in chemical forms; however, the correlation coefficient value obtained in each system suggests that this is not the case, at least for NQ and 2NQ. The case of 5NQ will be discussed below.

Based on Equations (2)–(6), isotherm parameters for all three systems are reported in Table 2. The R^2^ values suggest the best fit for the isotherm’s adsorption is the Freundlich model, where a multilayer of NQ molecules can be adsorbed onto SBA-15, and the best adsorption capacity (K_f_) is presented in the 2NQ@SBA-15 system at 32.2276 mg/g with moderate adsorption (n ≈ 1) [78]. These results are in agreement with those reported by other authors for the adsorption of prednisolone by SBA-15 [79] and meloxicam [80] and another system’s adsorption as famotidine by Zinc chloride particles [81].

As reported before, we would expect that 2NQ and 5NQ were deposited onto the SBA-15, while NQ preserves dispersion; however, the results indicate that according to the expected outcome, the best adsorption occurs between 2NQ and SBA-15 but not with 5NQ, as its adsorption capacity is even lower than NQ’s capacity. Therefore, we decided to evaluate these systems using computational chemistry techniques to understand the involved molecular mechanisms.

### 3.4. Theoretical Evaluation of the Adsorption Mechanism of NQ Derivatives on SBA-15

To further understand the interaction between NQ molecules and SBA-15 particles at the atomic level, we have used the density functional theory, B3LYP/6-31G+(d,p), in combination with the QTAIM topology analysis to study the behavior of these systems.

#### 3.4.1. Molecular Model Optimization Calculation

**Figure 6:**
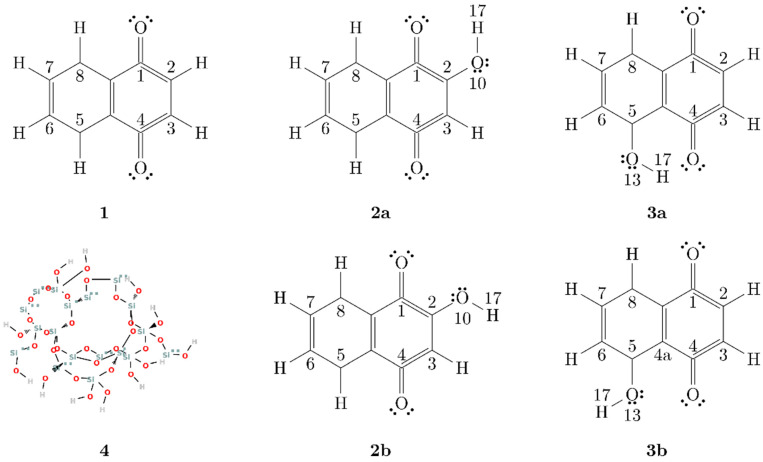
Structure of NQ derivatives and SiO_2_ molecules. (**1**) NQ; (**2a**) closed and (**2b**) open configuration of 2NQ; (**3a**) closed and (**3b**) open configuration of 5NQ; (**4**) SiO_2_ molecular model.

We constructed molecular models for NQ, 2NQ, and 5NQ based on the literature; several authors reported that NQs possess the ability to form intramolecular hydrogen bonds between their carbonyl and hydroxyl groups [82,83]. To consider this possibility, we constructed two models for the hydroxy-substituted NQs: closed structures (C) and open structures (O). In the closed-structure models, the hydroxyl group is oriented towards the carbonyl group, representing intramolecular hydrogen bridge-type interactions (structures 2a, 3a in Figure 6); in contrast, the open-structure models represent NQs without intramolecular hydrogen bond formations (structures 2b and 3b in Figure 6).

**Figure 7:**
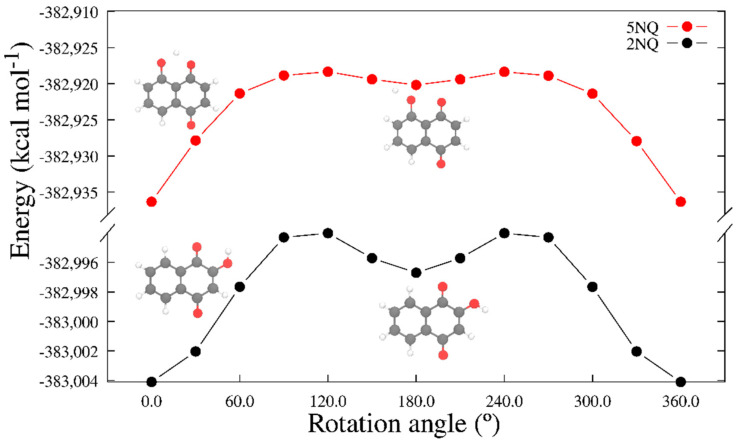
Energy obtained along the rotation of the dihedral angles C1-C2-O10-H13 for 2NQ and C4a-C5-O13-H17 for 5NQ.

To choose the more representative molecular structure of hydroxy-substituted NQ, we performed a potential energy scan toward the rotation of the dihedral angle C1-C2-O10-H13 for 2NQ and angle C4a-C5-O13-H17 for 5NQ. Figure 7 shows the closed structures at 0° and open structures at 180° for 2NQ and 5NQ. The structures of minima energy were those with intramolecular hydrogen bonds (closed structures).

#### 3.4.2. Calculation of the Interaction Energy (ΔE)

**Figure 8:**
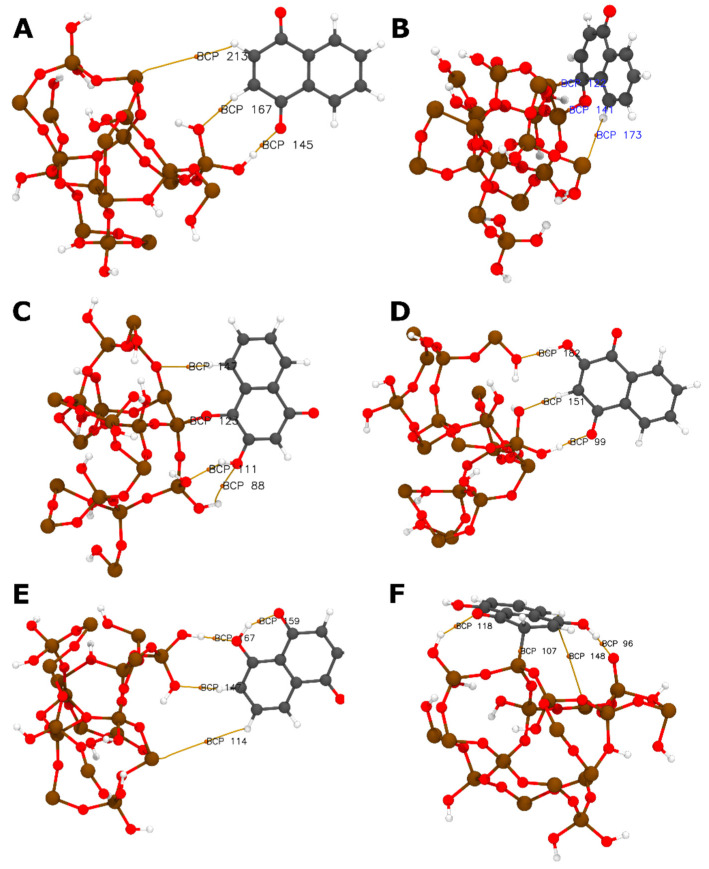
NQs@SiO_2_ interaction molecular models. (**A**) Horizontal (H) and (**B**) vertical (V) interactions of NQ; (**C**) closed horizontal (C.H.) and (**D**) open horizontal (O.H.) configuration of 2NQ; (**E**) closed horizontal (C.H.) and (**F**) open horizontal (O.H.) configuration of 5NQ. Black labels are the bond critical point (BCP) indices. Yellow lines correspond to bond paths.

The molecular interaction models were constructed from the optimized NQs and SiO_2_. Since NQs are planar molecules, we built interaction models by superimposing the NQ molecule in horizontal (H) and vertical (V) positions for the SiO_2_ surface. The open and closed structures of 2NQ and 5NQ were superimposed only in a horizontal position to the SiO_2_ surface. To calculate the interaction energy between the molecular models of NQs and the surface’s molecular model, we used Equation (7):

(7) $$\Delta E=E_{interaction}-\left(E_{surface}+E_{NQ}\right)$$

where $E_{interaction}$ is the interaction energy of the NQ@SiO_2_ system (Kcal mol^−1^), $E_{surface}$ is the SiO_2_ energy (Kcal mol^−1^), and $E_{NQ}$ is the NQs energy (Kcal mol^−1^). This interaction of energy parameter represents the total energy of the physical or chemical interactions involved in the binding or formation of the adsorption product. Table 3 presents ΔE, where we can observe that the interaction between SiO_2_ and NQ is stronger in the vertical position (Figure 8B) than in the horizontal position (Figure 8A). For 2NQ, there are no significant differences in the interaction for the closed system (Figure 8C) and the open system (Figure 8D). In contrast, a significant difference was observed for 5NQ, where the open system’s (Figure 8F) interaction is stronger than the closed system (Figure 8E).

The values corresponding to the three strongest interactions are as follows: (1) −96.08 kcal mol^−1^ and it corresponded to the interaction between vertical NQ (V) and SiO_2_. As observed in Figure 8B, this interaction is mediated by an H-bond-type interaction and two covalent interactions. One of these covalent interactions occurred between the silicon atom on the surface and the C4 of NQ, which probably promotes a change in C4’s hybridization from sp2 to sp3, as evidenced by a change in the dihedral angle (C8a-C4a-C4-O12; see Figure 6) from 180° in the sp2 configuration to 34° in the model interaction. (2) At −88.92 kcal mol^−1^ between the open horizontal 5NQ (O.H.) and SiO_2_ (Figure 8F), in this interaction, we can see two H-bond-type interactions and one covalent interaction between the C3 of 5NQ and a silicon atom on the surface. Additionally, we can observe that the C1 carbonyl group’s substrate obtains a proton from the surface, forming a hydroxyl group at C1. This could explain the modification of the crystalline pattern shown on XRD (see Figure 2D). Finally, (3) at −18.99 kcal mol^−1^ between the closed horizontal 2NQ (C.H.) and SiO_2_ (Figure 8C), we can see three H-bonds and one covalent interaction between the C1 carbonyl group of 2NQ and a silicon atom on the surface.

As can be seen, in all three systems with the strongest interaction energies, at least one covalent interaction is involved. To evaluate the nature of these bonds, we calculate the Mayer (MBO) [84] and fuzzy (FBO) [85] bond orders. The power of these parameters to investigate bonding in organometallic compounds has been widely demonstrated [86]. The value of MBO agrees with the empirical bond order; for single, double, and triple bonds, the values are close to 1.0, 2.0, and 3.0, respectively. On the other hand, the magnitude of FBO is comparable to MBO, especially for low-polar bonds, but it is more stable concerning the change in the basis set; therefore, we are more confident in the FBO values, as observed in Table 4. In the table, the bond order values obtained, especially the FBO values, are very close to one, evidencing the presence of a single covalent bond.

**Figure 9:**
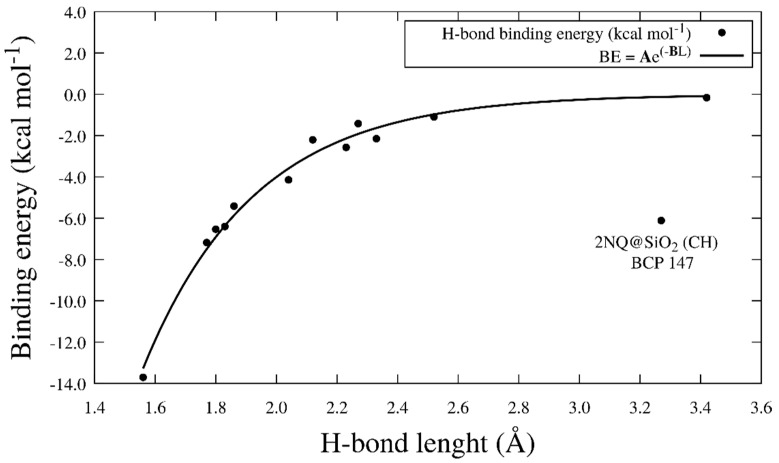
H−bond length and BE correlation.

Additionally, the H-bond binding energy (BE) was calculated with Equation (8), which is proposed initially by Emamian and collaborators [87]. Here, ρ_rBCP_ is the electron density at the bond critical point (BCP) corresponding to the H-bond obtained from the topological analysis:

(8) $$BE\approx -223.08\times \rho_{rBCP}+0.7423$$

where the unit of *ρ* is a.u., and the unit of BE is kcal mol^−1^. In Table 3, we show the BE calculated at every BCP obtained from the topology analysis of the interaction models. Figure 8 shows the NQ@SiO_2_, 2NQ@ SiO_2_, and 5NQ@ SiO_2_ interaction models and the BCP and paths from QTAIM. The QTAIM study confirms the intermolecular hydrogen bond between NQs and SiO_2_ for all systems, with 5NQ being in a horizontal position relative to SiO_2_ (Figure 8E,F); it is the system with the strongest hydrogen bond interaction (see Table 3). To relate the length of the H-bond to its binding energy, we plotted L_H_ vs. BE (Figure 9) and fit the data to the mathematical model proposed in Equation (9):

(9) $$BE=Ae^{-BL_{H}}$$

where *BE* = binding energy (kcal mol^−1^); L_H_ = H-bond length (Å); ***A*** = −929.5 kcal mol^−1^ is the energy at L_H_ = 0 Å; and ***B*** = 2.72 Å is the critical length at which the strength of the H-bond is too weak (R = 0.89. This model shows that the BE is stronger at small values of L_H_ and decreases to a critical length of 2.72 Å. The strongest H-bond occurs for the BCP96 at the new hydroxyl formed between the closed 5NQ and SiO_2_ surfaces (B.E. = −13.7 kcal mol^−1^), where L_H_ is 1.56 Å. In agreement with Buemi and Zucrello, the H-bond could be weak L_H_ > 2.2 Å, moderate 1.5 Å < L_H_ < 2.2 Å, and strong 1.2 Å < L_H_ < 1.5 Å [88].

## 4. Conclusions

The present work provided evidence that NQ derivatives follow a Freundlich isotherm adsorption model by either physical or chemical interactions. The 2NQ derivative has been found as the molecule with the highest adsorption capacity (32.2 mg g^−1^) with no considerable SBA-15 particle aggregations. The experimental findings are explained by DFT studies that show that 2NQ’s physisorption is mediated by H-bonds, and it is the system with more H-bond interactions. Covalent interactions can also be presented in NQ@SBA-15, 5NQ@SBA-15, and 2NQ@SBA-15 systems. These interactions can promote the deformation of NQ rings, and in the case of 5NQ, they can substrate a hydrogen atom from the surface of SiO_2_ and, thus, modifying the surface of SiO_2_, which is proved by a change in the XRD pattern. We believe that for 5NQ@SBA-15, covalent interactions decrease the adsorption capacity (1.8 mg g^−1^). In contrast, a stronger interaction occurs between the carbonyl group of naphthoquinones and the surface of SiO_2_. The results of this work establish a basis for elaborating a more robust adsorption/computational model that allows obtaining information on the interactions between molecules with antimicrobial actions and the surface of SBA-15 particles; consequently, this can improve the adsorption’s capacity based on the adsorbate chemical’s structure and the physicochemical characteristics of the particle. Our findings also contribute to understanding the cargo’s chemical structure and the adsorbent material in optimizing the design of drug-delivery systems, which will ultimately increase the probability of therapeutic success and decrease the related adverse effects in preclinical and clinical trials.

## Figures and Tables

**Table 1:** Characteristics of the SBA-15 and systems NQ@SBA-15, 2NQ@SBA-15, and 5NQ@SBA-15 obtained by X-ray diffraction (XRD), dynamic light scattering (DLS), z-potential measurements, and nitrogen adsorption/desorption isotherms.

Sample	Crystallite Size	Hydrodynamic Diameter	Z Potential	Surface Area	Pore Volume	Pore Width
	(Å)	(nm)	(mV)	(m^2^ g^−1^)	(cm^3^ g^−1^)	(nm)
SBA-15	911.23	618	−19.40	597.25	0.936	6.17
NQ@SBA-15	669.33	2301	−25.17	434.88	1.15	8.57
2NQ@SBA-15	814.66	667	−28.40	441.70	1.16	8.70
5NQ@SBA-15	743.52	3576	−22.97	438.99	1.14	8.84

**Table 2:** Langmuir, Freundlich, and Temkin isotherm parameters obtained by non-linear fittings for systems NQ@SBA-15, 2NQ@SBA-15, and 5NQ@SBA-15.

	Langmuir			Freundlich			Temkin		
	*Q*_0_	L	R^2^	*K*_*F*_	N	R^2^	*A*_*T*_	*B*_*T*_	R^2^
	(mg/g)	(dm^3^/mg)		(mg/g)			(L/g)		
**NQ @ SBA-15**	3.9746	0.0390	0.9576	6.3010	0.1394	0.9592	0.2251	175.6193	0.4083
**2NQ @ SBA-15**	51,590.5	0.0009	0.9836	32.2276	0.9071	0.9847	0.0767	1.0405	0.8289
**5NQ @ SBA-15**	3417.4	0.0024	0.9719	1.8176	0.7174	0.9797	2.0352	0.0281	0.9071

**Table 3:** Interaction energy (ΔE ), length of H-bond (L_H_), bond critical point (BCP) index, all electron densities at BCP (ρ_rBCP_), and H-bond binding energy (BE) values obtained from interaction models.

H-Bond		L_H_	BCP	ρ_rBCP_	BE
Donnor	Acceptor	(Å)		(Ha)	kcal mol^−1^
**NQ@SiO_2_ (H)**			**(** **Δ** **E = −10.74 kcal mol^−1^)**		
**SiO_2_−** **O_35_**	**H_76_−** **NQ**	2.23	145	0.0149	−2.57
**NQ−** **O_74_**	**H_53_−** **SiO_2_**	1.80	167	0.0326	−6.53
**NQ@SiO_2_ (V)**			**(** **Δ** **E = −96.08 kcal mol^−1^)**		
SiO_2_−Si_15_	H_80_−NQ	3.42	173	0.0034	−0.16
**2NQ@SiO_2_ (CH)**			**(** **Δ** **E = −18.99 kcal mol^−1^)**		
**SiO_2_−** **O_49_**	**H_80_−** **2NQ**	3.27	147	0.0307	−6.11
**2NQ−** **O_76_**	**H_53_−** **SiO_2_**	2.52	88	0.0083	−1.10
**SiO_2_−** **O_35_**	**H_81_−** **2NQ**	2.04	111	0.0219	−4.14
**2NQ@SiO_2_ (OH)**			**(** **Δ** **E = −16.06 kcal mol^−1^)**		
**SiO_2_−** **O_16_**	**H_81_−** **2NQ**	1.83	182	0.0320	−6.40
**SiO_2_−** **O_14_**	**H_75_−** **2NQ**	2.33	151	0.0130	−2.15
**2NQ−** **O_73_**	**H_59_−** **SiO_2_**	1.77	99	0.0355	−7.18
**5NQ@SiO_2_ (CH)**			**(** **Δ** **E = −8.95 kcal mol^−1^)**		
**5NQ−** **O_75_**	**H_81_−** **5NQ**	1.66	159	0.0492	−10.24
**5NQ−** **O_80_**	**H_53_−** **SiO_2_**	1.86	167	0.0276	−5.41
**SiO_2_−** **O_35_**	**H_77_−** **5NQ**	2.27	147	0.0097	−1.42
**5NQ@SiO_2_ (OH)**			**(** **Δ** **E = −88.92 kcal mol^−1^)**		
**SiO_2_−** **O_35_**	**H_55_−** **5NQ**	1.56	96	0.0648	−13.70
**5NQ−** **O_73_**	**H_59_−** **SiO_2_**	2.12	118	0.0132	−2.20

L = Bond length; BCP = bond critical point; ρ_rBCP_ = density of all electrons; BE = binding energy; CH = closed horizontal; OH = open horizontal.

**Table 4:** Interaction energy (ΔE), length of covalent bond (L_C_), Mayer bond order (MBO), and fuzzy bond order (FBO) values obtained from interaction models.

Bond		L_C_	MBO	FBO
		(Å)		
**NQ@SiO_2_ (V)**		**(Δ** **E = −96.08 kcal mol^−1^)**		
SiO_2_−Si_48_	C_69_−NQ	2.01	0.6365	0.7950
NQ−O_74_	Si_36_−SiO_2_	1.69	0.1579	1.3143
**2NQ@SiO_2_ (C.H.)**		**(** **Δ** **E = −18.99 kcal mol^−1^)**		
NQ−O_74_	Si_36_−SiO_2_	1.73	0.9474	1.1935
**5NQ@SiO_2_ (O.H.)**		**(** **Δ** **E = −88.92 kcal mol^−1^)**		
SiO_2_−O_11_	C_72_−NQ	1.89	0.7912	0.9522

## Data Availability

Not applicable.

## Supplementary Materials

The following supporting information can be downloaded at: https://www.mdpi.com/article/10.3390/ph15121464/s1.

## References

[1] Naimi T., Ringwald P., Besser R., Thompson S., Bell D. (2001). Antimicrobial resistance. Emerg. Infect. Dis..

[2] Yap J.K.Y., Tan S.Y.Y., Tang S.Q., Thien V.K., Chan E.W.L. (2021). Synergistic antibacterial activity between 1,4-naphthoquinone and β-lactam antibiotics against methicillin-resistant staphylococcus aureus. Microb. Drug Resist..

[3] Song R., Yu B., Friedrich D., Li J., Shen H., Krautscheid H., Huang S.D., Kim M.H. (2020). Naphthoquinone-derivative as a synthetic compound to overcome the antibiotic resistance of methicillin-resistant *S. aureus*. Commun. Biol..

[4] Ravichandiran P., Masłyk M., Sheet S., Janeczko M., Premnath D., Kim A.R., Park B.H., Han M.K., Yoo D.J. (2019). Synthesis and antimicrobial evaluation of 1,4-naphthoquinone derivatives as potential antibacterial agents. ChemistryOpen.

[5] Ibis C., Ozsoy-Gunes Z., Tuyun A.F., Ayla S.S., Bahar H., Stasevych M.V., Musyanovych R., Komarovska-Porokhnyavets O., Novikov V. (2016). Synthesis, antibacterial and antifungal evaluation of thio- or piperazinyl-substituted 1,4-naphthoquinone derivatives. J. Sulfur Chem..

[6] Estolano-Cobián A., Noriega-Iribe E., Díaz-Rubio L., Padrón J.M., Brito-Perea M., Cornejo-Bravo J.M., Chávez D., Rivera R.R., Quintana-Melgoza J.M., Cruz-Reyes J. (2020). Antioxidant, antiproliferative, and acetylcholinesterase inhibition activity of amino alcohol derivatives from 1,4-naphthoquinone. Med. Chem. Res..

[7] Goleva T.N., Lyamzaev K.G., Rogov A.G., Khailova L.S., Epremyan K.K., Shumakovich G.P., Domnina L.V., Ivanova O.Y., Marmiy N.V., Zinevich T.V. (2020). Mitochondria-targeted 1,4-naphthoquinone (SkQN) is a powerful prooxidant and cytotoxic agent. Biochim. Biophys. Acta—Bioenerg..

[8] McCall R., Miles M., Lascuna P., Burney B., Patel Z., Sidoran K.J., Sittaramane V., Kocerha J., Grossie D.A., Sessler J.L. (2017). Dual targeting of the cancer antioxidant network with 1,4-naphthoquinone fused gold(i) N-heterocyclic carbene complexes. Chem. Sci..

[9] Clementino-Neto J., da Silva J.K.S., de Melo Bastos Cavalcante C., da Silva-Júnior P.F., David C.C., de Araújo M.V., Mendes C.B., de Queiroz A.C., da Silva E.C.O., de Souza S.T. (2022). In vitro antitumor activity of dialkylamine-1,4-naphthoquinones toward human glioblastoma multiforme Cells. New J. Chem..

[10] Shen X.B., Wang Y., Han X.Z., Sheng L.Q., Wu F.F., Liu X. (2020). Design, synthesis and anticancer activity of naphthoquinone derivatives. J. Enzym. Inhib. Med. Chem..

[11] Widhalm J.R., Rhodes D. (2016). Biosynthesis and molecular actions of specialized 1,4-naphthoquinone natural products produced by horticultural plants. Hortic. Res..

[12] Ravichandiran P., Sheet S., Premnath D., Kim A.R., Yoo D.J. (2019). 1,4-Naphthoquinone analogues: Potent antibacterial agents and mode of action evaluation. Molecules.

[13] Pereyra C.E., Dantas R.F., Ferreira S.B., Gomes L.P., Silva F.P. (2019). The diverse mechanisms and anticancer potential of naphthoquinones. Cancer Cell Int..

[14] Wang Y., Luo Y.H., Piao X.J., Shen G.N., Meng L.Q., Zhang Y., Wang J.R., Li J.Q., Wang H., Xu W.T. (2019). Novel 1,4-naphthoquinone derivatives induce reactive oxygen species-mediated apoptosis in liver cancer cells. Mol. Med. Rep..

[15] Ishihara Y., Ishii S., Sakai Y., Yamamura N., Onishi Y., Shimamoto N. (2011). Crucial role of cytochrome p450 in hepatotoxicity induced by 2,3-dimethoxy-1,4-naphthoquinone in rats. J. Appl. Toxicol..

[16] Mitchell M.J., Billingsley M.M., Haley R.M., Wechsler M.E., Peppas N.A., Langer R. (2021). Engineering precision nanoparticles for drug delivery. Nat. Rev. Drug Discov..

[17] Patra J.K., Das G., Fraceto L.F., Campos E.V.R., Rodriguez-Torres M.D.P., Acosta-Torres L.S., Diaz-Torres L.A., Grillo R., Swamy M.K., Sharma S. (2018). Nano based drug delivery systems: Recent developments and future prospects. J. Nanobiotechnol..

[18] Alkahtani S., Alarifi S., Aljarba N.H., Alghamdi H.A., Alkahtane A.A. (2022). Mesoporous SBA-15 silica–loaded nano-formulation of quercetin: A probable radio-sensitizer for lung carcinoma. Dose-Response.

[19] Kazemzadeh P., Sayadi K., Toolabi A., Sayadi J., Zeraati M., Chauhan N.P.S., Sargazi G. (2022). Structure-property relationship for different mesoporous silica nanoparticles and its drug delivery applications: A review. Front. Chem..

[20] Žid L., Zeleňák V., Almáši M., Zeleňáková A., Szücsová J., Bednarčík J., Šuleková M., Hudák A., Váhovská L. (2020). Mesoporous silica as a drug delivery system for naproxen: Influence of surface functionalization. Molecules.

[21] Gkiliopoulos D., Tsamesidis I., Theocharidou A., Pouroutzidou G.K., Christodoulou E., Stalika E., Xanthopoulos K., Bikiaris D., Triantafyllidis K., Kontonasaki E. (2022). SBA-15 mesoporous silica as delivery vehicle for RhBMP-2 bone morphogenic protein for dental applications. Nanomaterials.

[22] Choi Y., Lee J.E., Lee J.H., Jeong J.H., Kim J. (2015). A biodegradation study of SBA-15 microparticles in simulated body fluid and in vivo. Langmuir.

[23] Zhao D., Huo Q., Feng J., Chmelka B.F., Stucky G.D. (1998). Nonionic triblock and star diblock copolymer and oligomeric sufactant syntheses of highly ordered, hydrothermally stable, mesoporous silica structures. J. Am. Chem. Soc..

[24] Fathi Vavsari V., Mohammadi Ziarani G., Badiei A. (2015). The role of SBA-15 in drug delivery. RSC Adv..

[25] Alazzawi H.F., Salih I.K., Albayati T.M. (2021). Drug delivery of amoxicillin molecule as a suggested treatment for COVID-19 implementing functionalized mesoporous SBA-15 with aminopropyl groups. Drug Deliv..

[26] Navarro-Tovar G., Palestino G., Rosales-Mendoza S. (2016). An overview on the role of silica-based materials in vaccine development. Expert Rev. Vaccines.

[27] Rasmussen M.K., Bordallo H.N., Bordenalli M.A., Akamatsu M.A., Trezena A.G., Tino-De-Franco M., Sant’Anna O.A., da Silva Martins T., de Souza Lopes J.L., de Abreu Fantini M.C. (2021). Assessing the efficiency of SBA-15 as a nanocarrier for diphtheria anatoxin. Microporous Mesoporous Mater..

[28] Rathinavel S., Ekambaram S., Korrapati P.S., Sangeetha D. (2020). Design and fabrication of electrospun SBA-15-incorporated PVA with curcumin: A biomimetic nanoscaffold for skin tissue engineering. Biomed. Mater..

[29] Li K., Sun H., Sui H., Zhang Y., Liang H., Wu X., Zhao Q. (2015). Composite mesoporous silica nanoparticle/chitosan nanofibers for bone tissue engineering. RSC Adv..

[30] Ugliengo P., Sodupe M., Musso F., Bush I.J., Orlando R., Dovesi R. (2008). Realistic models of hydroxylated amorphous silica surfaces and MCM- 41 mesoporous material simulated by large-scale periodic B3LYP calculations. Adv. Mater..

[31] Tandon H., Chakraborty T., Suhag V. (2019). A brief review on importance of DFT in drug design. Res. Med. Eng. Sci..

[32] Makkar P., Ghosh N.N. (2021). A review on the use of DFT for the prediction of the properties of nanomaterials. RSC Adv..

[33] Bader R.F.W. (1985). Atoms in molecules. Acc. Chem. Res..

[34] Oliveira B.G., Pereira F.S., de Araújo R.C.M.U., Ramos M.N. (2006). The hydrogen bond strength: New proposals to evaluate the intermolecular interaction using DFT calculations and the AIM theory. Chem. Phys. Lett..

[35] Najafi M., Morsali A., Bozorgmehr M.R. (2019). DFT study of SiO_2_ nanoparticles as a drug delivery system: Structural and mechanistic aspects. Struct. Chem..

[36] Grau E.N., Román G., Compañy A.D., Brizuela G., Juan A., Simonetti S. (2019). Surface modification vs sorption strength: Study of nedaplatin drug supported on silica. Appl. Surf. Sci..

[37] Noseda Grau E., Román G., Juan J., Compañy A.D., Simonetti S. (2021). Advance on adsorption of amino-functionalized silica nanocarrier for the delivery of therapeutic ampicillin as drug model. Inorg. Chem. Commun..

[38] Luo S., Hao J., Gao Y., Liu D., Cai Q., Yang X. (2019). Pore size effect on adsorption and release of metoprolol tartrate in mesoporous silica: Experimental and molecular simulation studies. Mater. Sci. Eng. C.

[39] Rafati A.A., Ebadi A., Bavafa S., Nowroozi A. (2018). Kinetic study, structural analysis and computational investigation of novel xerogel based on drug-PEG/SiO2 for controlled release of enrofloxacin. J. Mol. Liq..

[40] Masoumi M., Jahanshahi M., Ahangari M.G., Darzi G.N. (2020). Density functional theory study on the interaction of chitosan monomer with TiO_2_, SiO_2_ and carbon nanotubes. Mater. Chem. Phys..

[41] Abràmoff M.D., Magalhães P.J., Ram S.J. (2004). Image processing with ImageJ. Biophotonics Int..

[42] Schneider C.A., Rasband W.S., Eliceiri K.W. (2012). NIH image to ImageJ: 25 years of image analysis. Nat. Methods.

[43] Degen T., Sadki M., Bron E., König U., Nénert G. (2014). The high score suite. Powder Diffraction.

[44] Dabrowski A. (2001). Adsorption—From theory to practice. Adv. Colloid Interface Sci..

[45] Limousin G., Gaudet J.P., Charlet L., Szenknect S., Barthès V., Krimissa M. (2007). Sorption isotherms: A review on physical bases, modeling and measurement. Appl. Geochem..

[46] Chaker M. (2008). Applicability of some statistical tools to predict optimum adsorption isotherm after linear and non-linear regression analysis. J. Hazard. Mater..

[47] El-khaiary M.I. (2008). Least-squares regression of adsorption equilibrium data: Comparing the options. J. Hazard. Mater..

[48] Dennington R.D., Keith T.A., Milan J.M. (2008). Gauss View 5.0 2008.

[49] López L.I., Leyva E., de la Cruz R.F.G. (2011). Naphthoquinones: More than natural pigments. Rev. Mex. Ciencias Farm..

[50] Dera P., Lazarz J.D., Prakapenka V.B., Barkley M., Downs R.T. (2011). New insights into the high-pressure polymorphism of SiO_2_ cristobalite. Phys. Chem. Miner..

[51] Larsen A.H., Mortensen J.J., Blomqvist J., Castelli I.E., Christensen R., Dułak M., Friis J., Groves M.N., Hammer B., Hargus C. (2017). The atomic simulation environment—A python library for working with atoms. J. Phys. Condens. Matter.

[52] Becke A.D. (1993). Density-functional thermochemistry. III. The role of exact exchange. J. Chem. Phys..

[53] Spitznagel G.W., Clark T., Chandrasekhar J., Schleyer P.V.R. (1982). Stabilization of methyl anions by first-row substituents. The superiority of diffuse function-augmented basis sets for anion calculations. J. Comput. Chem..

[54] Hariharan P.C., Pople J.A. (1973). The influence of polarization functions on molecular orbital hydrogenation energies. Theor. Chim. Acta.

[55] Frisch M.J., Trucks G.W., Schlegel H.B., Scuseria G.E., Robb M.A., Cheeseman J.R., Scalmani G., Barone V., Mennucci B., Petersson G.A. (2009). Gaussian 09. Wallingford CT.

[56] Lu T., Chen F. (2012). Multiwfn: A multifunctional wavefunction analyzer. J. Comput. Chem..

[57] Niculescu V.C., Paun G., Parvulescu V. (2018). New organometallic complex supported on mesoporous silica and its enzymes activity inhibition properties. Appl. Organomet. Chem..

[58] Tahiri N., Khouchaf L., Elaatmani M., Louarn G., Zegzouti A., Daoud M. (2014). Study of the thermal treatment of SiO_2_ aggregate. IOP Conference Series: Materials Science and Engineering.

[59] Scherrer P. (1912). Bestimmung der inneren struktur und der größe von kolloidteilchen mittels röntgenstrahlen. Kolloidchemie Ein Lehrbuch.

[60] Patterson A.L. (1939). The scherrer formula for X-ray particle size determination. Phys. Rev..

[61] Fiorilli S., Onida B., Bonelli B., Garrone E. (2005). In situ infrared study of SBA-15 functionalized with carboxylic groups incorporated by a co-condensation route. J. Phys. Chem. B.

[62] Esperanza Adrover M., Pedernera M., Bonne M., Lebeau B., Bucalá V., Gallo L. (2020). Synthesis and characterization of mesoporous SBA-15 and SBA-16 as carriers to improve albendazole dissolution rate. Saudi Pharm. J..

[63] Zinicovscaia I., Yushin N., Humelnicu D., Grozdov D., Ignat M., Demcak S., Humelnicu I. (2021). Sorption of Ce(Iii) by silica Sba-15 and titanosilicate Ets-10 from aqueous solution. Water.

[64] Aranda-López Y., López-López L., Castro K.E.N., Ponce-Regalado M.D., Becerril-Villanueva L.E., Girón-Pérez M.I., Del Río-Araiza V.H., Morales-Montor J. (2021). Cysticidal effect of a pure naphthoquinone on taenia crassiceps cysticerci. Parasitol. Res..

[65] Rivera-Ávalos E., de Loera Carrera D., Araujo-Huitrado J.G., Escalante-García I.L., Muñoz-Sánchez M.A., Hernández H., López J.A., López López L. (2019). Synthesis of amino acid–naphthoquinones and in vitro studies on cervical and breast cell lines. Molecules.

[66] Silveira G.Q., Ronconi C.M., Vargas M.D., Gil R.A.S.S., Magalhães A. (2011). Modified silica nanoparticles with an aminonaphthoquinone. J. Braz. Chem. Soc..

[67] Sun D., Kang S., Liu C., Lu Q., Cui L., Hu B. (2016). Effect of zeta potential and particle size on the stability of SiO_2_ nanospheres as carrier for ultrasound imaging contrast agents. Int. J. Electrochem. Sci..

[68] Wang P., Keller A.A. (2009). Natural and engineered nano and colloidal transport: Role of zeta potential in prediction of particle deposition. Langmuir.

[69] Rouquerol J., Avnir D., Everett D.H., Fairbridge C., Haynes M., Pernicone N., Ramsay J.D.F., Sing K.S.W., Unger K.K. (1994). Guidelines for the characterization of porous solids. Stud. Surf. Sci. Catal..

[70] Thommes M., Kaneko K., Neimark A.V., Olivier J.P., Rodriguez-Reinoso F., Rouquerol J., Sing K.S.W. (2015). Physisorption of gases, with special reference to the evaluation of surface area and pore size distribution (IUPAC technical report). Pure Appl. Chem..

[71] Norsuraya S., Fazlena H., Norhasyimi R. (2016). Sugarcane bagasse as a renewable source of silica to synthesize santa barbara amorphous-15 (SBA-15). Procedia Eng..

[72] Deryło-Marczewska A., Zienkiewicz-Strzałka M., Skrzypczyńska K., Świątkowski A., Kuśmierek K. (2016). Evaluation of the SBA-15 materials ability to accumulation of 4-chlorophenol on carbon paste electrode. Adsorption.

[73] Langmuir I. (1917). The constitution and fundamental properties of solids and liquids. II. Liquids. J. Am. Chem. Soc..

[74] Langmuir I. (1917). The constitution and fundamental properties of solids and liquids. J. Frankl. Inst..

[75] Freundlich H. (1907). Über die adsorption in lösungen. Z. Phys. Chem..

[76] Chen H., Zhao J., Dai G., Wu J., Yan H. (2010). Adsorption characteristics of Pb(II) from aqueous solution onto a natural biosorbent, fallen *Cinnamomum camphora* leaves. Desalination.

[77] Temkin M.I. (1940). Kinetics of ammonia synthesis on promoted iron catalysts. Acta Physiochim. URSS.

[78] Yao Y., Bing H., Feifei X., Xiaofeng C. (2011). Equilibrium and kinetic studies of methyl orange adsorption on multiwalled carbon nanotubes. Chem. Eng. J..

[79] Albayati T.M., Jassam A.A.A. (2019). Synthesis and characterization of mesoporous materials as a carrier and release of prednisolone in drug delivery system. J. Drug Deliv. Sci. Technol..

[80] Alkafajy A.M., Albayati T.M. (2020). High performance of magnetic mesoporous modification for loading and release of meloxicam in drug delivery implementation. Mater. Today Commun..

[81] Abniki M., Azizi Z., Panahi H.A. (2021). Design of 3-aminophenol-grafted polymer-modified zinc sulphide nanoparticles as drug delivery system. IET Nanobiotechnol..

[82] Armendáriz-Vidales G., Martínez-González E., Cuevas-Fernández H.J., Fernández-Campos D.O., Burgos-Castillo R.C., Frontana C. (2013). The stabilizing role of intramolecular hydrogen bonding in disubstituted hydroxy-quinones. Electrochim. Acta.

[83] Vega-Rodríguez S., Jiménez-Cataño R., Leyva E., Loredo-Carrillo S.E. (2013). Intramolecular hydrogen bonds in fluorinated, methoxylated, or unsubstituted 2-(anilino)-1,4-naphthoquinones. A theoretical study. J. Fluor. Chem..

[84] Mayer I. (1983). Charge, bond order and valence in the AB initio SCF theory. Chem. Phys. Lett..

[85] Mayer I., Salvador P. (2004). Overlap populations, bond orders and valences for “fuzzy” atoms. Chem. Phys. Lett..

[86] Bridgeman A.J., Cavigliasso G., Ireland L.R., Rothery J. (2001). The mayer bond order as a tool in inorganic chemistry. J. Chem. Soc. Dalton Trans..

[87] Emamian S., Lu T., Kruse H., Emamian H. (2019). Exploring nature and predicting strength of hydrogen bonds: A correlation analysis between atoms-in-molecules descriptors, binding energies, and energy components of symmetry-adapted perturbation theory. J. Comput. Chem..

[88] Buemi G., Zuccarello F. (2004). DFT study of the intramolecular hydrogen bonds in the amino and nitro-derivatives of malonaldehyde. Chem. Phys..

