# Supplementary material for: Interactions of Antibacterial Naphthoquinones with Mesoporous Silica Surfaces: A Physicochemical and Theoretical Approach
Source: Pharmaceuticals (Basel). 2022 Nov 25;15(12):1464. doi: 10.3390/ph15121464 (PMC9787537; doi:10.3390/ph15121464)
Supplement: Supplementary file 1 [file pharmaceuticals-15-01464-s001.zip › pharmaceuticals-2033106-supplementary.pdf]

Suplementary material

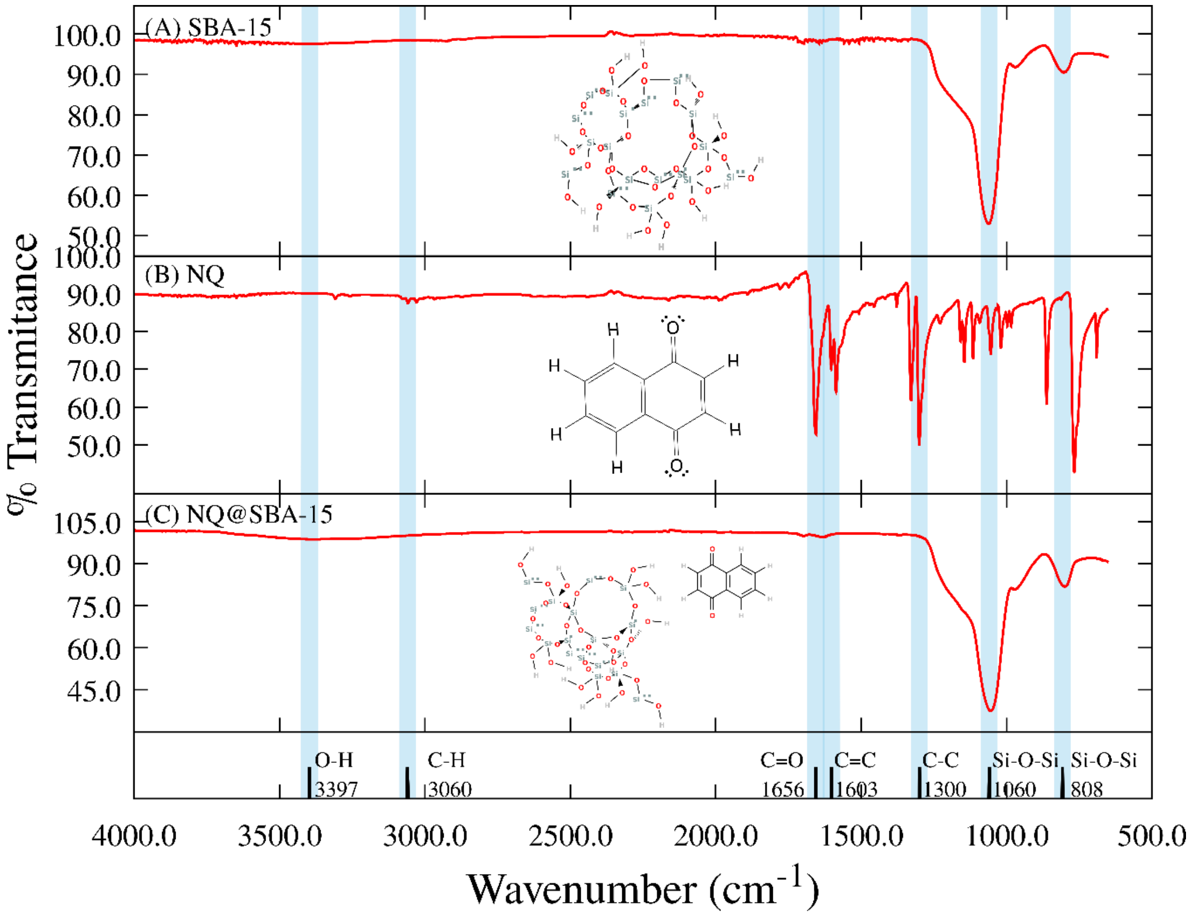

Figure S1. FTIR spectra from top to bottom, (A) SBA-15, (B) NQ, and (C) NQ@SBA-15 interaction.

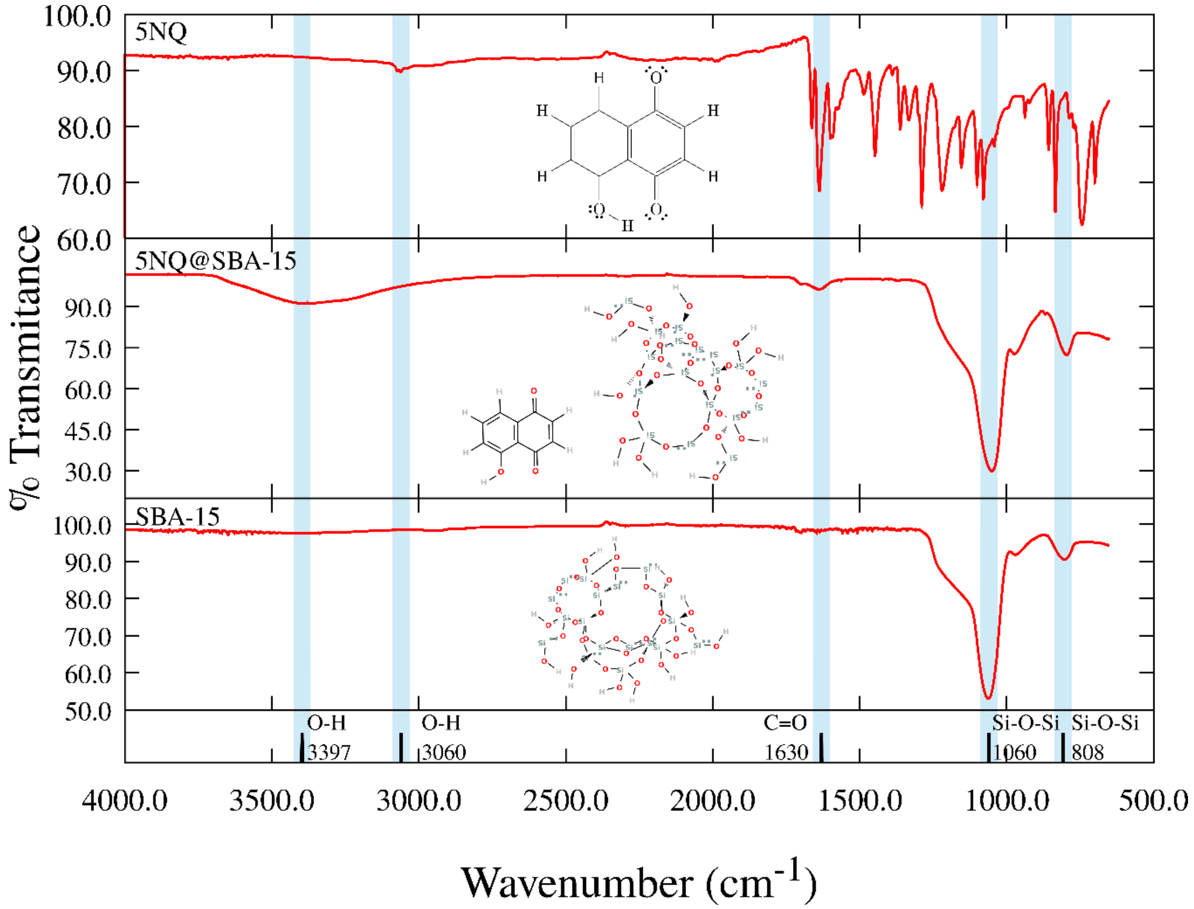

Figure S2. FTIR spectra from top to bottom SBA-15, 5NQ, and 5NQ@SBA-15 interaction.
